# Guided Tissue Regeneration in Heart Valve Replacement: From Preclinical Research to First-in-Human Trials

**DOI:** 10.1155/2015/432901

**Published:** 2015-10-01

**Authors:** L. Iop, G. Gerosa

**Affiliations:** Department of Cardiac, Thoracic and Vascular Sciences, University Hospital of Padua, Via Giustiniani 2, 35128 Padua, Italy

## Abstract

Heart valve tissue-guided regeneration aims to offer a functional and viable alternative to current prosthetic replacements. Not requiring previous cell seeding and conditioning in bioreactors, such exceptional tissue engineering approach is a very fascinating translational regenerative strategy. After *in vivo* implantation, decellularized heart valve scaffolds drive their same repopulation by recipient's cells for a prospective autologous-like tissue reconstruction, remodeling, and adaptation to the somatic growth of the patient. With such a viability, tissue-guided regenerated conduits can be delivered as off-the-shelf biodevices and possess all the potentialities for a long-lasting resolution of the dramatic inconvenience of heart valve diseases, both in children and in the elderly. A review on preclinical and clinical investigations of this therapeutic concept is provided with evaluation of the issues still to be well deliberated for an effective and safe in-human application.

## 1. Introduction

Cardiac diseases (CVD), particularly heart valvulopathies, dramatically affect the world population representing in 2012 the primary cause of death for 17.5 millions of people. This number is expected to almost duplicate by 2030; thus, the World Health Organization is prompting a sustained programme for effective CVD prevention, management, and monitoring [1–3].

Characterized by rheumatic, degenerative, or genetic etiology, heart valve diseases interest both pediatric and adult patients and at end-stage, they require surgical intervention of reconstruction of the ventricular outflow tract, as well as correction of its components to restore the unidirectionality of blood flow among the heart chambers.

Standard healthcare procedures foresee the implantation of valve prostheses of either mechanical or biological composition, for which life-long anticoagulation, inability to repair/grow, or structural deterioration is considered the main hindrance for a long-lasting functional treatment in cardiopathic patients. Among the current valve prostheses, allogeneic valvulated conduits, commonly named homografts, represent the most akin replacement to a native tissue, even if their availability is seriously limited by organ donation shortage. Despite the high structural and functional resemblance, valve homografts have been reported to degenerate in nearly 15 years, like their animals-derived counterparts submitted to xenoimmunological shielding through glutaraldehyde treatment. At the basis of heart valve bioprosthetic degeneration are recipient's inflammatory and immune responses directed against allo-/xenogeneic epitopes, as, for example, the human leukocyte antigens (HLA) II or animal immunological triggers.

In the search for the effective replacement, a close similarity to the native valve is desirable. Adequate hemodynamic profile, facilitated diffusion of oxygen and nutrients, tissue adaptation to somatic growth, and full biocompatibility in terms of absent inflammatory/immune stimuli and thrombogenic power are pursued in the design and manufacturing processes of the ideal valve substitute [4] by application of tissue engineering principles. A multidisciplinary strategy is addressed to the development and creation of viable surrogate tissues for a prospective replacement of malfunctioning valves. Synthetic or natural scaffolds are engrafted with differentiated or stem cells and submitted to dynamic stimulation in bioreactors, conceived to simulate the complex mechanical and chemical prompts able to generate a physiological tissue/organ maturation [5].

Synthetic polymers, such as polylactic and polyglycolic acids (PLA and PGA resp.), have been the first scaffolds applied for tissue engineered heart valves (TEHVs). Thanks to the tunable porosity and bioadsorbability, these biopolymers facilitate cell infiltration and likely* de novo* deposition of extracellular matrix (ECM) by the same engrafting elements. Cell type selection for TEHVs has been heterogeneous. From differentiated cells, as saphenous vein-derived endothelial and fibroblast cells, to progenitors or less committed elements, as mesenchymal or endothelial progenitor cells, a strong propensity for adhesion and/or tissue integration has been demonstrated [6–8]. Specifically, accurate studies on heart valve embryonic developmental steps fostered the implementation of the manufacturing process with cues essential for a boosted cell differentiation [9–11]. A dynamic conditioning for at least 14 days was reported to be sufficient for effective maturation of cell-repopulated synthetic scaffolds prior to implantation.

While several technical advancements have been proposed for* in vitro* formulation and* in vivo* delivery of such TEHVs, specific flaws still hamper their clinical applicability. Inability to reach mature composition, distribution, and conformation of newly synthesized ECM, in terms of collagen, glycosaminoglycans, and furthermore elastin network, prevents the achievement of proper hemodynamic functionality in the right circulation system, where these valve prototypes are usually tested. Improper valve stratification and cusp thickening provoke incompetent leaflet coaptation and, hence, regurgitation events, which could be expected to worsen in conditions of arterial circulation.

Conversely, a natural scaffold has already reached the state of maturation of its extracellular matrix fibers. After effective removal of endogenous cellular elements by means of enzymatic and/or detergents-based decellularization, it can be implemented as a starter matrix for further colonization with selected cell types.

Apart from classical TEHVs, a novel valve substitute with* bona fide* self-regeneration ability has been advanced, namely, the tissue-guided regenerated replacement.

This review paper aims to evaluate the evolution steps of heart valve tissue-guided regeneration from the first bench experiments to the outcomes of the current bed applications.

## 2. Which Scaffold? and Which Decellularizing Manipulation?

The concept of tissue-guided regeneration has been first introduced with reference to the medical branches of dental and bone bioengineering, by implying the implantation of biomimetic scaffolds able to stimulate themselves the regeneration process without further factors or biological signals [12]. In sharp distinction to the classical tissue engineering conception, it does not contemplate the creation* in vitro* or* in situ* of a living tissue, but the induction of healing and adaptive remodeling through host's cell engraftment and natural physiological conditioning (body as a bioreactor). Conceived, instead, by many researchers as an exception or a peculiar modality of tissue engineering, it has been applied more recently to other medical fields, as in cardiovascular regenerative medicine.

The combination of cells and synthetic scaffolds has not be sufficient itself to reproduce the complexity of a vital and functional 3D tissue. In order to drive effective homing and instruct cells to proper differentiation and integration, scaffolds should ideally possess also matrikine signals and adhesion proteins typical of a healthy native tissue. Matrikines are classified as extracellular matrix domains able to interact with specific cell surface molecules belonging to the large family of cytokines, chemokines, and growth factors. In concert with adhesion receptors, for example, ligands to integrins, these proteins significantly influence cell proliferation, migration, and differentiation [13]. Composition and 3D spatial distribution of such domains in the starting matrices are thus fundamental for successful healing processes and discriminate a biomaterial with self-regenerating abilities from a scaffold leading to immature or pathological tissue formation [10, 14, 15].

Full reverse engineering of a native valve tissue ECM by artificial manufacturing is still a remote prospect. Outflow tract heart valves are, in fact, highly complex structures with intrinsic biological and biomechanical properties granting their opening and closure for virtually 3 billion times during the entire lifespan [10, 16].

Biological assets, as ECM anisotropy and its dependent cell distribution and specializations, as well as precise biomechanical characteristics, as specific coaptation height and minimum leaflet flexion, influence functionality and durability of native aortic valves.

Natural heart valves have been implanted in replacement surgery since the 1970s, by adopting animal tissues earlier submitted to glutaraldehyde treatment for immunologic shielding and ECM stabilization [17]. With the pioneering surgical procedure introduced by Ross and colleagues [18, 19], valve homografts entered the full clinical stage, being applied as replacement of the autologous pulmonary root, resected and heterotopically implanted in aortic position. Unfortunately, longevity of these valves in the patients is poor for mid-/long-term occurrence of structural deteriorations.

Many hypotheses have been advanced in relation to bioprosthetic degeneration, as, for instance, the higher mechanical stress introduced by the surgical implantation or the lack of viable cells induced during glutaraldehyde chemical treatment or after cryopreservation [20, 21]. In particular, the cytotoxicity of glutaraldehyde and its free aldehyde groups provokes in treated cells firstly a suffering state and then their death with extracellular space release of phosphate groups, as phospholipids, in the form of the so-called matrix vesicles, and nucleic acids that act as powerful nucleation sites for calcification [21]. To improve the stability of glutaraldehyde-treated tissues, several technical adjustments have been attempted. Organic solvents, for example, opportune mixtures of ethanol, octanediol, and/or octanol, have been applied during bioprosthetic valve manufacturing to extract cell membrane phospholipids, as well as to crosslink collagen-elastin network [22]. Moreover, detoxification processes have been introduced to neutralize free aldehyde groups of glutaraldehyde. These chemical agents are indeed particularly toxic for their ability to interact with primary amino groups of proteins or nitrogen atoms in DNA, giving rise to Schiff bases. In addition, they are able to trap plasmatic calcium further contributing to the triggering or amplification of calcific events [23, 24]. Amino acids and amino compounds, that is, glycine, L-glutamic acid, and/or urazole, have been used as free aldehyde groups neutralizers [24, 25].

Current efforts are posed on the discovery of novel and less cytotoxic crosslinking agents: as an example, porcine heart valves treated with the natural gardenia fruit compound genipin were observed conserving a cell viability around 79–100% with effective induction of tissue stability [26, 27].

Through a different mechanism, cell viability is threatened in cryopreserved allografts by the formation of ice crystals during the cryocooling phase with induced mechanical stress, intercellular crystallization and thermal shock, and/or by the cytotoxic effect of osmotic phenomena during the thawing phase [28]. Controlled settings of temperature decrease (−1°C/min) and cryoprotector inclusion (DMSO or glycerol) are nowadays utilized in tissue banks for the effective storage of allografts before their clinical need.

Despite the optimizations operated, experimental evidences demonstrate that such modifications are not sufficient for the complete protection of allografts and xenografts from structural deterioration. A consistent set of data has been more and more demonstrating that immunological issues are the triggering factors in the onset of tissue degeneration. Biochemical and histological analyses on 7 commercially available animal-derived valve replacements revealed that only one bioprosthesis, that is, the Epic TM valve from St. Jude Medicals (St. Paul, MN), has a complete shielding of alpha-gal xenoantigens. Such xenogeneic epitopes are oligosaccharides of glycoproteins and glycolipids, displayed on the surface of vascular endothelial and stromal cells in all mammals except apes, Old World monkeys, and humans. An evolutionary gene silencing of the enzyme alpha 1,3-galactosyltransferase renders these species unable to metabolize alpha-gal [29, 30]. Retrieved at a concentration of at least 10^7^ epitopes per porcine cell, alpha-gal is also expressed by the human gut bacterial flora, kept under control by the 1% serum circulating specific IgGs [8, 29–33].

Several studies correlated alpha-gal to the priming of heart valve calcification through an immune response mechanism [34]. The incomplete masking of the epitope could be exacerbated* in vivo* by the tissue fatigue, to which the valve is submitted in the patient's arterial circulation. The new crosslinker genipin is per se able to only attenuate the inflammatory and immune responses to xenogeneic tissues [26]. Moreover, non-gal xenoantigens have been identified and no clinical information is yet available regarding their impact on bioprosthetic graft survival.

The theoretically more biocompatible human valve allografts fail, however, in a similar time to the xenogeneic counterparts. Again, the main culprit of structural deterioration is the immune reaction against donor's antigens [35]. In particular, the endothelium has been considered to behave as antigen presenting cell element to the host's immune system [36]. The presence of HLA II (or HLA DQ/DR) has been related to humoral and cellular responses preventing any accommodation or tolerance of the graft and chronically inducing its rejection [37, 38].

A modality to render allo-/xenogeneic matrices immunoprivileged and possibly susceptible to further autologous-like regeneration has been offered by decellularization technology. This extraction process has as rational the removal of all endogenous cell elements, as, for instance, cell membranes, organelles, and nucleic acids, which can adversely prompt inflammatory, immune, and calcific events [39, 40].

Several methods for decellularization of organ and tissues have been formulated, mainly based on osmotic shock, detergents, and/or enzymes in possible combination with mechanical agitation and heat to facilitate tissue exposure to the chemicals.

A treatment with hypotonic or hypertonic solutions or their alternation, such as low and/or high ionic strength solutions, can easily induce cell lysis. Hypertonic stress is severely destructive for several proteins, including those of the contractile cell machinery, by causing their aggregation and misfolding in treated tissues and organs [41]. EDTA or other chelating agents are frequently added in the first decellularization steps to disrupt cell-cell binding and potentiate the action of the following treatments [42].

Ionic detergents, as sodium dodecyl sulfate (SDS), act very harshly on protein-protein interactions, while also solubilizing cell membranes of nuclear type [43]. Nonionic detergents, such as Triton X-100, show mild solubilization: their main targets are the lipids in interaction with themselves or with proteins [44].

Enzymes are usually applied to disrupt cell-cell binding and proteins. They are eventually used as a terminal step of the extraction process to remove any trace of nucleic acids released from lysed cells: such nucleases operate the hydrolysis of the interior or exterior bonds of ribo- and deoxyribonucleotide chains, otherwise closely sticking to the ECM fibers after release in the extracellular space [8].

Most commonly applied procedures relying on SDS and/or on the trypsin enzyme demonstrated efficacy in the decellularizing yield but, in turn, revealed inadequate preservation of the valvular ECM. Elastin fragmentation, collagen swelling, or, more generally, ECM denaturation and precipitation were frequently observed. Furthermore, these chemical treatments affect the glycosaminoglycan moiety of hyaluronan, cheratins and chondroitin sulfates [14]. As a result, the tertiary and quaternary structures of associated ECM macromolecular aggregates are altered and it is destabilized the ability to appease the shocks, provoked by the enormous pressure variations during the entire cardiac cycle [45–47].

Among ionic detergents, bile acids and their salt anions do not cause protein denaturation and, hence, are more conservative for treated ECMs. Sodium cholate and deoxycholate find large application in the decellularization procedures in combination with either Triton X-100 (TRICOL) [8, 47] or SDS [48], as well as alone [49]. They differ from SDS for the rigid steroidal groups in their backbone [43]. Less frequently, zwitterionic detergents, characterized by mixed properties of anionic and nonionic surfactants, are applied, but with several detrimental effects [50]. Biochemical and ultrastructural evaluations of most scaffolds decellularized with bile acids-based treatments revealed good preservation of collagen and elastin network. Nevertheless, different results in terms of glycosaminoglycan and basal membrane integrity were evidenced. Collagen IV, proteoglycans, and glycosaminoglycans composing the basal* laminae* are minimally affected by sodium cholate [6, 8, 51], while they can be damaged and largely removed by sodium deoxycholate [52]. The conservation of the original basal membrane is essential for further endothelial cell adhesion and for the prevention of platelet attachment/activation once* in vivo*.

Zhou and colleagues investigated the effects of different decellularizing treatments on ECM preservation, as well as on thrombogenicity and immunogenicity after* in vitro* direct contact with human blood. No ECM disruption and complete decellularization were observed with sodium deoxycholate. Among all proposed methods, including also the use of SDS, trypsin/EDTA, or trypsin-detergent-nuclease, thrombogenic and immunological responses to sodium deoxycholate-treated ECMs were surely higher [53]. A confirmation to these observations was obtained through a quantitative methodology based on immunoblotting technique: Arai and Orton demonstrated that a conspicuous amount of soluble protein antigens were still detectable in bovine pericardium and porcine heart valves after decellularization with SDS and sodium deoxycholate [54]. On the contrary, the treatment with sodium deoxycholate alone for 48 hours revealed reducing smooth muscle actin to 0.96 ± 0.71%, as well as the total soluble protein to 6.68 ± 2.0% [52].

## 3. Preclinical Proof-of-Concept of Heart Valve Tissue-Guided Regeneration

In the large panorama of* in vivo* preclinical TEHV testing, the researchers using decellularized scaffolds as starting matrices challenged the application of the sole ECM with respect to its standard arrangement with cells.

With a technology named SynerGraft (CryoLife, Atlanta, Georgia), O'Brien et al. were able to manufacture acellular porcine valve conduits by means of a decellularization treatment based on hypotonic solutions, enzymes for the removal of nucleic acids, and extensive washout. When implanted in sheep, the xenografts demonstrated competence for 6 months and, at* ex vivo* analysis, fibroblast repopulation interested leaflet stroma [55]. Similar results were obtained in the same ovine model with human cryopreserved decellularized heart valves in both aortic and pulmonary orthotopic positions [56].

The second heart valve tissue-guided regeneration trial was realized in pulmonary position by Konertz and colleagues. By comparison of the standard TEHV procedure to its exception, they implanted allogeneic sodium deoxycholate-decellularized valves, either nude or previously cell-seeded, to reconstruct the right ventricle outflow tract in juvenile sheep animals. The outcomes were surprisingly in favor to the sole scaffold implantation, as documented by regenerative processes initiated in the valve allografts at 6 months and by the increase in the annulus diameter in response to somatic growth, reported at nearly 1-year follow-up [57, 58].

As a further step towards the clinical stage, this group performed a comparative assessment of cryopreserved allografts versus decellularized heterografts (pig) in the sheep model for a time interval of 10 months. In a simulated Ross intervention approach, hemodynamic function of xenografts revealed to be satisfactory. In addition, sheep pulmonary homografts appeared partially devitalized in some valvular regions, while the acellular porcine valves (from now on referred with the proprietary name Matrix P) were free from calcifications and repopulated by endothelial cells and fibroblasts [59]. In recent times, aortic valve xenografts decellularized with the same technology were implanted orthotopically in juvenile sheep. After 4 months of evaluation in systemic circulation, no adverse events in terms of hemodynamic and biological performance were disclosed [60]. In another preclinical study in the systemic circulation, porcine aortic valves decellularized with sodium deoxycholate were implanted stented in an allogeneic model. Decellularized valves were well performing and prone to recellularization without calcified foci, which were revealed instead for the controls, that is, Carpentier-Edwards glutaraldehyde-treated commercial bioprostheses (Edwards Lifesciences, Irvine, CA) [61].

Allogeneic aortic valve conduits were evaluated by Baraki et al. in an ovine model after decellularization with sodium deoxycholate and SDS. Implanted in orthotopic position, such valves were trivially regurgitant and did not exhibit degeneration or dilatation after 9 months of* in vivo* follow up. While fresh native allografts used as control showed signs of mineralization, deterioration, and advanced insufficiency already at 3 months, decellularized aortic valves were interested by minimal calcification and incipient repopulation [62].

The ability to prevent calcification introduced in heart valves by decellularization was also described by Hopkins and colleagues in an allogeneic sheep model: calcium deposits were absent from acellular cryopreserved pulmonary valve allografts, whereas severely concerned their native analogues [63].

With the rational to test acellular aortic valves as substitutes for the reconstruction of the right ventricle outflow tract, we manufactured porcine valvulated conduits by applying TRICOL technology. Allogeneic decellularized valves were implanted in a porcine model for maximum 15 months, revealing a good hemodynamic performance over time. TRICOL decellularized substitutes exhibited* ex vivo* progressive regeneration without signs of degeneration (calcifications, fibrosis, and/or thrombosis) or immune rejection. Original ECM architecture was well preserved. Engrafted cell elements displayed native-like features, as the ability to synthesize novel ECM (collagen and elastin fibers). Regenerated valves appeared vascularized by newly formed, mature blood vessels, that is, capillaries and* vasa vasorum*, especially in* media* and* adventitia* of arterial walls. Interestingly, hallmarks of reinnervation were documented. Regenerative processes were also confirmed by the presence of reparative macrophages, namely, M2 populations, in higher amount with respect to proinflammatory M1. Stem cell elements of embryonic, hematopoietic, neural, and mesenchymal lineages populated the regenerated aortic valves in specific topographic regional localization. Such regeneration signs further confirm the ability of the decellularized native ECM to behave as a stem cell niche providing cues for proper cell differentiation [64].

## 4. Heart Valve Tissue-Guided Regeneration in the Clinical Arena

Despite the initial difficulties in the acceptance of this experimental concept as therapeutic, this is the unique TEHV modality apart from solely endothelialized scaffolds to have reached to date translational applicability in heart valve diseased patients.

CryoLife designed, patented, and distributed two novel valve bioprostheses with the same SynerGraft decellularization technology based on osmotic shock: 500/700 and CryoValve biodevices.

After the positive outcomes observed in 6 months with 500/700 SynerGraft porcine valves in a weanling sheep right ventricular outflow tract reconstruction model [55], Simon et al. implanted these valvular substitutes in 4 pediatric patients. A catastrophic rapid failure of the xenografts led to 3 deaths in less than 1 year and a prophylactic valve substitution in the unique surviving child. At bioptic examination, a strong lymphocyte reaction was documented. In the disclosure study, the causes for such hyperacute and acute rejections were attributed to the xenogeneic extracellular matrix [65], even if possible culprits should have been researched in residual xenoantigens (see* Alpha-gal: the nightmare in xenogeneic valve implantation *in Section 5).

The other commercial option by CryoLife, that is, CryoValve, is a decellularized valve allograft. Following the data provided by the company, CryoValve has been already implanted in 5.700 patients since 2000 [66]. The biodevice was first evaluated in a small cohort of 36 individuals, where adequate functionality and no panel reactive antibody response were documented after 3 months from surgery [67].

In a comparison to historical controls, 14 pediatric patients, implanted with CryoValve for reconstruction of cardiac valve defects, displayed decreased titers of HLA I and II alloantibodies, when tested at 1, 3, and 12 months postoperatively [68]. Comparable outcomes were disclosed by Zehr et al. after aortic orthotopic implantation of the same cryopreserved decellularized allogeneic valve in 22 adult patients (range 31–80 years) [69].

A reduced titer of panel reactive antibodies is very advantageous especially for patients awaiting heart transplantation.

In 2005, Sayk et al. described histopathological findings regarding an explanted pulmonary Cryovalve from a 60-year-old patient who died for bronchopneumonia. After 5 weeks from implantation, no signs of degeneration could be appreciated. Indeed, coaptation was optimal without any leaflet thickening. At microscopic examination, polymorphonucleocytes and macrophages were found migrated in the proximal and distal sutures and involved in the elimination of nonviable donor myocardium. No progenitor, fibroblast, or myofibroblast infiltration was observed [70]. Such aspects conform to the outcomes described by Elkins and colleagues with reference to the two-phase progression of CryoValve regeneration in the ovine xenogeneic model [56].

After having received US FDA approval in 2008, CryoValve was adopted in many cardiosurgical centers rendering possible trials on noninferiority evaluation with respect to standard cryopreserved allografts. No postsurgery mortality, freedom from redo procedures, and lower incidence of severe regurgitation were valued in this comparison [71, 72].

Espousing both the classical and tissue-guided regeneration TEHV conceptions, Dr. Haverich and his research group started a clinical trialing with human pulmonary valve allografts, decellularized in 2002 with enzymatic (trypsin) treatment and from 2009 with sodium deoxycholate/SDS detergents. In contrast to conventional cryopreserved allografts and glutaraldehyde-fixed bovine jugular vein valves, decellularized fresh allogeneic valves (manufactured from 2006 within the company Corlife oHG, Hannover, Germany) were characterized by improved freedom from explantation, lower transvalvular gradients, and ability to adaptive growth. Moreover, immune responses mediated by T lymphocytes and natural killers were absent during the early-term evaluation of 3.5 years [73–75].

Analogously, Dohmen and colleagues evaluated the functional performance of acellular allogeneic valves, both alone or submitted to previous seeding with patient's vascular cells. From 2003 to 2007, 68 patients underwent Ross procedure with implantation of a pulmonary decellularized allograft. The patients' group was subdivided in equal number and submitted to implantation of either SDS- or sodium deoxycholate-decellularized pulmonary valves. Adequate valve performance was recorded in a 4-year-long follow-up with a low mortality rate. A discrete increase of transvalvular gradients was observed over time for control allografts and allogeneic valves decellularized with sodium deoxycholate, while no modification was reported for SDS-treated ones [76]. An immunological and echocardiographic evaluation was performed by the same group on sodium deoxycholate-decellularized allografts by comparison to the cryopreserved correspondents. A total of 20 patients were examined at specific frequency from surgery. In particular, HLA alloantibodies titers and eventually panel reactive antibody levels were determined at 5, 10, 30, 90, and 180 days postoperatively. In patients implanted with cryopreserved allografts, marked value elevations of HLA I and HLA II were documented already after 30 post-surgery days. Contrasting data were reported for decellularized allografts: nearly 60% of patients displayed no increased values, while for the rest of them either modest HLA I rise or abnormal panel reaction antibody levels were documented [77].

In the 2005–2010 lustrum, da Costa et al. implanted in aortic position 41 allogeneic heart valves decellularized with SDS. Submitted to arterial circulation, acellular valves demonstrated patency and low rates of calcification. One of treated patients underwent reoperation due to incoming mitral stenosis. During surgery, a small fragment of the implanted aortic conduit was excised. The histopathologic analysis of the aortic wall sample revealed intact ECM, intimal hyperplasia, and trivial medial repopulation by fibroblast-like cells [78].

A clinical trial with allogeneic aortic heart valves treated with TRICOL technology decellularization is now ongoing.

Apart from valvular allografts, another xenogeneic acellular valve, that is, Matrix P, has been tested in clinical applications. As aforementioned, Matrix P is a porcine pulmonary heart valve decellularized through the sodium deoxycholate treatment. The technology has been developed and tested by Dohmen and colleagues and is commercially distributed by Autotissue Ltd. (Berlin, Germany). A variant of the Matrix P with equine pericardial patch extension, that is, Matrix P Plus, has been conceived for the surgical cases, where a distal or proximal patch extension is required. Both valves were granted with the European CE mark in the 2004-2005 biennium.

Matrix P substitutes were first applied clinically from 2002 in 103 patients undergoing to the Ross procedure and subsequent hemodynamic evaluation (transthoracic echocardiography and eventually multislice computed tomography) at discharge, 3, 6, and 12 months after intervention. Two patients died after surgery for multiorgan sepsis and one reoperation was performed for false aneurysm after 10 months from intervention. In the explanted xenograft, endothelial cell lining and stromal engraftment of recipient's cells were evidenced without signs of inflammation or immune rejection [79].

From 2006 to 2008 (30.5 months), 61 pediatric and adult patients were submitted to right ventricle outflow tract reconstruction with Matrix P or Matrix P Plus. Early mortality, settled at almost 8%, was due to non-xenograft related causes, while postinterventional valve failure occurred in 4 cases, retreated with the same Matrix P Plus. An additional valve with adequate hemodynamics was removed after 1.5 years during reoperation of an unsolved, complex cardiac defect reconstruction. No degeneration aspects were observed at macroscopic examination and histological evaluation revealed absence of inflammation but reendothelialization of leaflets and intimal pulmonary wall. Normal structure and lack of calcification were documented in the remaining xenografts for the entire intermediate follow-up by computed tomography and magnetic resonance [80].

In another study, serum samples were collected from 159 patients who underwent heart valve replacement with glutaraldehyde-treated bioprostheses (porcine or bovine) or Matrix P/Matrix P Plus. With reference to preimplantation values, immunological potential of decellularized xenogeneic valves was evaluated immediately after surgery and at 9–12 months postoperatively. IgG and IgM titers against porcine collagen I and alpha-gal were investigated to evaluate possible immune reactions and identify their triggers in ECM or cellular elements. No significant alteration was reported for anticollagen response in all implants. Anti-gal titers were indeed raised in commercial biological prostheses with respect to decellularized ones, for which a sole IgM response was documented [81].

Divergent results were although disclosed after implantation of the same Matrix P valves in other independent studies [82–86]. According to the outcomes of these clinical evaluations, freedom from graft dysfunction ranged from 50 to 60% with stenosis and pseudoaneurysm as main failure signs and inflammatory and fibrotic processes as histopathological evidences.

The controversial results with SynerGraft and Matrix P grafts should impose more caution in the application of xenogeneic tissues-derived biodevices in humans. A step back to the preclinical evaluation in human-like models (non-human primates) is mandatory to verify effective biocompatibility in xenointeractions.

## 5. Future Developments

Tissue-guided regeneration concept in heart valve surgery evolved quite rapidly considering that the first preclinical experiments were performed around the middle 1990s and the first-in-human trials were carried out in 2000.

Allogeneic decellularized heart valves can now be considered as a functional therapeutic replacement option; however, data about their long-term clinical evaluation are still incomplete.

Decellularized valve xenografts have entered the clinical stage too early after nonrobust preclinical investigation.

Many aspects remain hitherto to ponder for a prospective considerate clinical suitability of heart valve tissue-guided regeneration.

### 5.1. Uncomfortable Leavings of Decellularization Treatments: Cytotoxicity Issues

In the formulation of the decellularization cocktail, particular attention should be addressed to the biochemical properties of each agent used. More precisely, change of these features is strictly dependent on variations in the working microenvironment (temperature, pH, light, etc.).

Enzymes, applied for disruption of cell-cell interactions or for digestion of nucleic acids, work in specific regimens of temperature and ionic strength, and prolonged exposure can induce serious damages to treated cardiovascular scaffolds [44].

Detergents exert their activity depending on the critical micelle concentration (cmc), namely, the minimum concentration for individual molecules to spontaneously cluster and form micelles. The cmc is influenced by previously cited microenvironmental conditions, as well as by the presence of proteins, lipids, and other detergents. High cmc is a characteristic of bile acids, as sodium cholate and deoxycholate, but also of ionic detergents with low concentration of counterions.

Membrane proteins are solubilized by detergents through a mimic of the natural lipid bilayer environment, in which the same ones are found. An elevated cmc level is particularly crucial in decellularization technology, since the detergents can be more easily washed out from the scaffolds. Variations in the pH and temperature of the working solution can reduce detergent solubility and, for instance, cause gelation states, precipitation, or phase separations [43, 87].

Insoluble detergents remain entrapped among the fibers of the decellularized ECM. Such condition induces a cytotoxic microenvironment preventing further cell colonization [42, 88].

### 5.2. Age of the Donor

Several changes in valvular ECM architecture and composition are induced by ageing, as shown by some elegant works [10, 14]. Such varying ECM and cell properties should be taken into account for TEHV design, especially for the approaches relying only on the instructing abilities of the scaffolds.

It is still unknown whether a decellularization treatment may induce different effects on a young or adult ECM, but it is likely that the extraction power could be superior in a still immature scaffold.

Moreover, considering allogeneic valvular implantation, the donor's average age is rising to more than 50 years, the age in which heart valve pathophysiological alterations are frequently observed as consequence of hypertension and/or hypercholesterolemia [89].

### 5.3. Preclinical Heart Valve Evaluation: The Need for Standardization

Animal studies are undeniably essential to test the risk management associated with novel (bio)devices and prevent therapeutic design errors in patients. Following ISO 5840 standards,* in vivo* studies for the assessment of a valve device need to be performed site-specifically in at least 10 animals for a duration of 20 weeks. Two sham animals have to be included for controls, even if there is no specification, about which already approved bioprostheses should be used on this purpose. Hemodynamic, hematochemical and pathological tests must be carried out to evaluate performance and signs of structural damage, calcification, thromboembolism, inflammation, and degenerative processes.

ISO regulations do not specify the animal species of the testing model. So far, preclinical models for heart valve prosthetic evaluation have included sheep and pig animals. The ovine model is the most utilized [55–63]. Juvenile sheep (3–6 months) are excellent models to investigate calcification propensity. These animals well simulate the human valve physiology for similar anatomy, annulus size, heart rate, and relatively slow and limited somatic ingrowth. Conversely, platelet activity is reduced with respect to humans [90]. In addition, the juvenile ovine model failed to predict hyperacute immune rejection in xenointeraction with decellularized porcine valves [55, 65].

Pigs are adequate to assess valve-associated thrombotic complications: in fact, their platelets show* in vitro* an equal activity to human ones [61]. Porcine breeds with lower growth rate are to be preferred to standard animals in order to properly simulate a human pediatric being [64, 90].

Other animal models, such as the dog and the non-human primate, find fewer applications in studies of heart valve assessment due to socio-ethical limitations associated with their use for scientific aims. However, it will be pivotal to evaluate biocompatibility of xenogeneic decellularized valves in a model closer to the human, such as the non-human primate.

Apart from hemodynamic studies, most histopathological analyses are confined to a modest investigation with poor identification of engrafting cell elements and their effective viability and activity in the regenerated tissue. As an example, smooth muscle actin is frequently adopted as a marker of smooth muscle but can be expressed also by activated myofibroblasts: thus, alone, it is not indicative of* bona fide* cell differentiation and maturation. Additionally, extensive molecular and ultrastructural investigations should be performed to fully dissect the regeneration process and clearly distinguish inflammatory and immune responses in implanted decellularized valves.

### 5.4. Alpha-Gal: The Nightmare in Xenogeneic Valve Implantation

Most decellularization methods reach an effective yield in the removal of all endogenous cell components. However, the extractive power of allo- or xenoantigens from human or animal valve tissues may vary considerably. Kasimir et al. suggested in 2005 that the elimination of alpha-gal xenoantigens could be achieved by adding as a further step to their sodium deoxycholate/Triton X-100-based decellularization a treatment with the nonionic, nondenaturing IGEPAL CA-630 detergent. This variation to the standard protocol was reported to be efficient in the removal of alpha-gal as proven at the histological level [91]. Recently, we demonstrated through a proprietary ELISA test based on a commercially available antibody against alpha-gal that the epitope amount is only halved in porcine heart valves submitted to sodium cholate, Triton X-100, and IGEPAL treatment [92]. Despite the application of a similar decellularization protocol, such different experimental outcomes are to be ascribed to the methodology applied for alpha-gal detection, that is, the use of a high affinity isolectin versus an extremely specific antibody [8]. IGEPAL effects on extracellular matrix biocompatibility were also investigated in a rat subdermal model. In contrast with untreated acellular ECMs, IGEPAL counterparts revealed a high propensity for neoangiogenesis (unpublished data). For a future clinical application in humans, such an aspect has to be considered as pathological.

At the present time, TRICOL method is the sole decellularization technology proven to manufacture valve xenoscaffolds completely deprived of alpha-gal [8, 33, 92].

Other immunological modifications are currently tested to abolish the alpha-gal burden. Transgenic pigs knock-out for this xenoantigen have been generated: hyperacute rejection of obtained hearts was prevented in immunosuppressed baboons for at least 6 months [93]. Additional treatments with recombinant alpha-1,3 galactosidase obtained from* Bacteroides thetaiotaomicron* demonstrated being effective in the removal of the xenoantigen from decellularized cardiovascular scaffolds [94, 95].

While these modifications are likely efficacious, they are associated with high technical complexity and expensive costs of production.

As already mentioned, alpha-gal is not the sole xenoantigen in animal-derived tissues, though its role in hyperacute rejection manifestations is undisputed. Non-gal antibodies are described as further barrier to xenotransplantation. A captivating bioengineering hypothesis has been formulated in recent times by Uri Galili in order to favour an immediate regeneration and hence avoid rejection mediated by non-gal antibodies. Nanoparticles loaded with alpha-gal could be co-injected in grafting procedures with the rational to bind anti-gal antibodies and force recruitment of pro-healing macrophages through the release of complement chemotactic factors, thus accelerating remodelling processes for a prompted autologous-like tissue formation. Positive preliminary results of this intriguing approach in fibrocartilage and ischemic myocardium settings have been lately disclosed [96–98]. Further confirmation and, especially, verification in other xenotransplantation challenges, such as the same heart valve xenointeraction, are attended. Potentially, such a tissue functionalization could be easily introduced in the manufacturing of decellularized heart valves for guided-tissue regeneration. A soak in a liquid medium containing these nanoparticles could be sufficient to allow diffusion through the collagenous network.

### 5.5. Increasing Biocompatibility, Bioactivity, and Calcification Protection: The Multifunctional Fillers

Biofunctionalization by means of hydrogel incorporation represents a smart modality to introduce regenerative signals or improve the biomechanical properties in the decellularized scaffold microenvironment. As suggested by Galili, the incorporation in hydrogel fillers of alpha-gal nanoparticles could allow for a homogenous distribution into treated scaffolds [98]. As space filler, Jeong et al. utilized polyethylene glycol, able to stabilize protein properties and protect them from degradation of proteolytic enzymes. This macromolecular substance demonstrated being superior in preventing calcification to polyacrylamide [99]. The same group developed a novel valve bioprosthesis based on a porcine tissue aortic root submitted to decellularization (SDS, Triton X-100, and sodium lauryl sarcosinate), *α*-galactosidase digestion, space filler functionalization, glutaraldehyde treatment, ethanol/octanol organic solvent, and glycine-mediated detoxification [100]. Although this extensively modified xenogeneic prosthesis demonstrated promising functionality and resistance to calcification both in an* in vitro* circulation experiment and in heterotopic (aortic-mitral) implantation in the sheep model, the utilization of glutaraldehyde excludes any valve remodeling and/or adaptation to the somatic growth of the recipient, that is, the key goals of tissue-guided regeneration.

### 5.6. Tissue Banking: The Need for Effective Procedures of Disinfection, Sterilization, and Preservation

In order to avoid the transmission of pathogenic contaminants and be distributed as sterile biodevices, decellularized valves need to be submitted to proper disinfection or sterilization methods.

A further advantage of decellularization procedures is given by the possibility to manipulate tissues in sterile settings without modifying the extraction power of the treatment.

Prior to allogeneic implantation, human decellularized scaffolds are currently submitted to similar antibiotic (/antimycotic) cocktails applied in tissue banks. Careful analyses should be accomplished to investigate the effects of residual antibiotics on scaffold repopulation. For instance, amphotericin B is known to modify cell differentiation in mononuclear cells, which are often participating in regenerative events* in vivo* [101].

Due to their animal origin, xenografts must be terminally sterilized before clinical application in order to eliminate any transmissible agent, such as bacteria, fungi, and species-specific viruses with human tropism (e.g., porcine endogenous retrovirus). While antibiotic solutions do not introduce any structural modification in treated valves, procedures of sterilization by *γ* irradiation, electron beam, or microwave alter significantly collagen, elastin, and other molecules of the ECM.

For valve bioprostheses, the guidelines contained in ISO 14160 standards indicate the application of terminal liquid sterilization (TLS) to ensure complete reset of the bioburden, that is, sterility assurance level of 10^−6^. The worst-case scenario has to be simulated also by means of superinfections to verify the efficacy of the applied TLS. After the sterilization treatment, valve biodevices must be unmodified in their hemodynamic and biocompatibility assets. Among the TLS liquids, glutaraldehyde and formaldehyde find most application in the manufacturing of valve prostheses. However, TLS by means of glutaraldehyde or formaldehyde does not maintain the bioactivity of treated tissues and it is associated with cytotoxicity induction as previously reported.

For decellularized allografts and xenografts, novel sterilization methodologies should be designed.

In an attempt of sterilization of heart valves, Chen and Wika patented in 2003 a method for the induction of tissue drying with glycerol followed by ethylene oxide treatment or ionizing radiation (Patent file US 6534004 B2). The application of non-thermal microwave radiation was reported by Shamis and colleagues to be less aggressive on ECM scaffolding but not able to generate complete sterilization [102]. A sterilant treatment with 22.5 kGy gamma irradiation provoked, instead, a severe destruction of the fibers of the ECM [103].

The storage of decellularized heart valves in tissue banks could render immediate the distribution to the cardiac surgery units as off-the-shelf replacements. So far, cryopreservation has found large use for the preservation of heart valve allografts but also of their decellularized equivalents. The suitability of this technique for proper valve storage is much debated. Synergraft cryopreserved decellularized allogeneic valves did not show signs of structural damage upon observation with transmission electron microscopy and two photon-laser scanning confocal imaging [104]. Similarly, we evidenced that no collagen swelling/shrinkage or clearly distorted/disrupted elastin fibers could be observed in TRICOL-decellularized cryopreserved pulmonary valve leaflets [8]. Schenke-Layland et al. reported conversely the onset of detrimental effects on ECM fibers after cryopreservation [105] and proposed an alternative treatment, that is, vitrification, as more conservative for effective preservation. Vitrification can be achieved by adding formamide and 1,2-propanediol to the standard cryopreservation method, based on the cryoprotectant DMSO. Heart valves treated with this methodology demonstrated good hemodynamic profile* in vivo* with respect to standard allografts [106].

Residuals of cryoprotectants, such as DMSO, formamide, and 1,2-propanediol, may induce an ECM milieu toxic for engrafting cells.

### 5.7. Bioengineering Tools to Improve Recellularization

Different from other cardiovascular tissues, heart valves can exert their hemodynamic function even devitalised or acellular. In the mid-/long-term, however, remodeling abilities are required to sustain somatic ingrowth in children or, in general, to operate reparative processes. Such tissue competences may rely only on functional cells.

As also evidenced by Zilla et al. in the context of vascular graft repopulation [107], regenerative abilities could vary from species to species, with the human less inherently prone to a fast resolution in healing events.

Histopathological findings of resected decellularized valves prove the slower onset of repopulation in humans with respect to the previously observed animal models [78–80]. To improve recellularization, biofunctionalization methodologies are currently explored via nanotechnological modifications. In particular, coating with DNA aptamers or antibody-mediated enhanced homing revealed being optimal routes to drive the repopulation with selected circulating cell elements [108, 109].

### 5.8. Socio-Economic Concerns upon Costs and Beneficial Effects for the Cardiopathic Population

Even if decellularized allo-/xenogeneic valve replacements are relatively less labor-intensive with regard to standard tissue-engineered heart valves, the costs for their purchase are prohibitive for the healthcare system.

Moreover, the implementation of the manufacturing process with further technical innovations (i.e., transgenic alpha-gal KO pigs, recombinant enzymes, etc.) may drastically increase the Industrial production expenditures.

Tissue-guided regenerated heart valves risk therefore being a therapeutic option for few. As observed by Burch et al. [110], the basin of patients eligible for this valve surgical treatment depends on a close evaluation of risks/benefits. In this viewpoint, long-term follow-up outcomes will shed definitely light on the effective superiority ranking of decellularized valves with respect to common allografts.

### 5.9. Demand for Specific Guidelines in Decellularized Valve Risk Assessments for Clinical Applicability Granting

The rapid evolution of heart valve tissue engineering has not been accompanied by a similarly fast legislative response. As previously highlighted in several points of this review, the regulation for a considerate clinical application of novel decellularized biodevices is to date incomplete. In the lack for specific guidelines, such novel bioreplacements are rendered equivalent to standard bioprostheses, for which, however, characteristics of viability or support to living cell elements are not contemplated.

As we already pointed out elsewhere, no regulations are still available for the clinical application of xeno-derived biomaterials. Already granted with US FDA approval or CE marking, many commercial biodevices distributed as decellularized are actually devitalized and with a strong xenoantigenic load harmful for treated patients [65, 111].

## 6. Conclusions

The timesaving and the relative simplicity in the manufacturing of acellular valve scaffolds render this therapeutic concept easily up scalable in Industrial productions and possibly available for a population of both diseased infants and adults. With all the prospects for a life-long durability and abilities of remodelling and adaptation, tissue-guided regenerated valvular conduits could drastically reduce the number of reinterventions and hence the health system costs for heart valve disease management.
